# Classification of facial paralysis based on machine learning techniques

**DOI:** 10.1186/s12938-022-01036-0

**Published:** 2022-09-07

**Authors:** Amira Gaber, Mona F. Taher, Manal Abdel Wahed, Nevin Mohieldin Shalaby, Sarah Gaber

**Affiliations:** 1 Systems and Biomedical Engineering Department, Faculty of Engineering, Cairo University, Giza, Egypt; 2 Neurology Department, Faculty of Medicine, Cairo University, Giza, Egypt; 3 Department of Neuromuscular Disorder and Its Surgery, Faculty of Physical Therapy, Cairo University, Giza, Egypt

**Keywords:** Facial paralysis, Grading, Ensemble classification, Machine learning, Kinect, Facial animation units

## Abstract

Facial paralysis (FP) is an inability to move facial muscles voluntarily, affecting daily activities. There is a need for quantitative assessment and severity level classification of FP to evaluate the condition. None of the available tools are widely accepted. A comprehensive FP evaluation system has been developed by the authors. The system extracts real-time facial animation units (FAUs) using the Kinect V2 sensor and includes both FP assessment and classification. This paper describes the development and testing of the FP classification phase. A dataset of 375 records from 13 unilateral FP patients and 1650 records from 50 control subjects was compiled. Artificial Intelligence and Machine Learning methods are used to classify seven FP categories: the normal case and three severity levels: mild, moderate, and severe for the left and right sides. For better prediction results (Accuracy = 96.8%, Sensitivity = 88.9% and Specificity = 99%), an ensemble learning classifier was developed rather than one weak classifier. The ensemble approach based on SVMs was proposed for the high-dimensional data to gather the advantages of stacking and bagging. To address the problem of an imbalanced dataset, a hybrid strategy combining three separate techniques was used. Model robustness and stability was evaluated using fivefold cross-validation. The results showed that the classifier is robust, stable and performs well for different train and test samples. The study demonstrates that FAUs acquired by the Kinect sensor can be used in classifying FP. The developed FP assessment and classification system provides a detailed quantitative report and has significant advantages over existing grading scales.

## Introduction

Facial paralysis (FP) is a loss of facial movements due to facial nerve pathology. It results in impairment of functions of voluntary facial muscles innervated by the facial nerve leading to facial asymmetry [1]. FP is clinically classified into two categories: peripheral or lower motor neuron (LMN), and central or upper motor neuron (UMN) [2]. Peripheral FP is a nerve disturbance in the pons of the brainstem. It affects the facial muscles in the lower, middle and upper regions of one facial side. Central FP (due to stroke) is a nerve dysfunction in the motor cortical areas, only the lower half of the face on one side is affected [3].

The majority of FP patients suffer from peripheral facial palsy. Since it affects most of the facial muscles of one side of the face, it is difficult for the patient to perform the normal movements of the eyes, eyebrows, and mouth.

The causes of peripheral FP are divided into four: idiopathic, traumatic, infectious, and neoplastic [4]. Idiopathic paralysis or Bell’s palsy is the common cause of peripheral FP.

The precise diagnosis and early treatment of FP helps in rapid improvement and recovery. There is currently no standardized clinical assessment for lower motor impairment and most of the available grading tests are subjective, time consuming and not applied in routine daily practice. An accurate, non-invasive, quantitative, and objective evaluation and classification system of FP is still required. Such a system is essential in selecting treatment and rehabilitation protocols as well as evaluating improvement in the follow-up phase.

### Current facial paralysis classification systems

The methods of extracting features which depend on facial asymmetry for FP classification are divided into two categories: hand-crafted features based or deep learning-based methods [5]. The hand-crafted methods depend on prior knowledge for extracting the facial asymmetrical features. On the other hand, deep learning-based methods can learn and automatically extract the palsy-specific features. One example of the deep learning approach [5] applied convolutional neural networks (CNNs) on FP images to automatically extract palsy-specific features. These features were then used to classify five FP grades.

Several works have employed machine learning using facial features for FP classification. In one work [6], an ensemble of regression trees was used for iris extraction and facial salient points on 2D images and were found to provide improved FP classification. The facial symmetry score is evaluated from the ratio of both iris area and the distances between certain facial landmarks in both sides of the face. Different classifiers (e.g., random forest, decision tree, etc.) were employed to classify between peripheral palsy (PP) and central palsy (CP).

In [1], a CNN model was used for FP classification using 2D images, and was found to achieve high accuracy compared to neurologists’ diagnosis. To reduce the subjectivity factor, the dataset was divided into seven categories: normal, left mild dysfunction, left moderate dysfunction, left severe dysfunction, right mild dysfunction, right moderate dysfunction, and right severe dysfunction. The triple-check approach was used in labeling of the dataset.

When using CNNs, overfitting may occur which means that there is a biasing towards the training set causing small training error and large testing error. This limitation of CNNs can be resolved by applying data augmentation. One study applies a generative adversarial network (GAN) to augment the training dataset by synthesizing face images with varying facial palsy grades [5].

In another work [7], a parallel hierarchy convolutional neural network (PHCNN) was developed to assess and classify FP and was applied to the publicly available databases: YouTube Facial Palsy (YFP) [8] and Extended CohnKanade (CK +) [9]. This method was able to distinguish between normal and FP subjects based on dividing the facial area into two palsy regions.

An approach was proposed to assess and classify the FP stage based on the analysis of facial skin perfusion from the facial blood flow image [10]. Facial blood flow distribution characteristics are extracted using an advanced segmentation technique. Three classifiers; K-nearest neighbor (K-NN), SVM, and Neural Network (NN) are then applied to provide a quantitative evaluation of FP based on the House–Brackmann scale.

In clinical assessment of FP, both the static facial asymmetry at different facial movements and the dynamic change of movement are considered. However, most of the current research uses only the asymmetrical facial features in FP evaluation. One research study presents an approach for automatic FP classification based on the static and dynamic features [11]. It is based on SVM in quantifying the static asymmetry and classifying the degrees of FP in each facial movement. The rate of features change in both sides of the face is used to evaluate the dynamic asymmetry.

In 2018, Banita and Tanwar [12] proposed an approach of classifying the FP severity into one of the three categories: patient can be cured, patient cannot be cured, and patient may or may not be cured. Based on the House–Brackmann system, grades II–V reflects patient with FP (can recover or not), whereas grade VI in House–Brackmann system reflects a patient with total FP who cannot recover. The methodology gives better accuracy based on the 3D images with the fuzzy logic.

Anguraj and Padma [13] developed a method for classifying the severity level of FP into three categories: mild, moderate, severe beside the normal case. First, Salient Point Selection Algorithm (SPSA) is used to assign a grade for facial movements. Then, Feed Forward Back Propagation Neural Network (FFBPN) is achieved to classify the severity of the disease. The few number of images (9 images) is one of the limitations of this study.

Table 1 summarizes a comparison of recent FP classification systems. The fields of comparison are the targeted facial movements, the traditional grading system considered as a ground truth, the tools and the machine learning algorithms used for the classification process, and the specifications of the dataset used. Also the limitations of each system are shown in the table.

**Table 1:** Comparison of recent FP classification systems

References	Objective	Facial movements	Ground truth	Tools	Dataset	Performance	Limitations
Chaoqun Jiang et al. 2020 [10]	FP classification (6 FP grades)		HB	LSCI scanners K-NN SVM NN	RGB images blood flow images 80 unilateral FP patients	Accuracy NN 96.77% K-NN 67.74% SVM 86.77%	
Xin Liu et al. 2020 [7]	FP classification (3 severity levels)	Rest Open mouth Closure the eyes lightly Elevation of eyebrows Pursing lips etc.	HB	PHCNN-LSTM	YouTube Facial Palsy Database Extended CohnKanade Database	Accuracy PHCNN-LSTM 0.9481%	Few public FP databases available Lack of various facial expressions in the datasets
Jocelyn Barbosa et al. 2019 [6]	Health classification (normal/patient) FP classification (PP/CP)	Rest Raising of eyebrows Screwing-up of nose Smiling with showing of teeth		RLR RF SVM DT NB Hybrid	440 2D images 60 normal subjects 40 PP patients 10 CP patients	Sensitivity RLR 85.9% RF 92.3% SVM 72.5% DT 90.2% NB 79.9%	No evaluation of FP degree No classification of facial paralysis grade Small dataset
Anping Song et al. 2018 [1]	FP classification (7 categories)	Rest Eye closed Eyebrows raised Cheeks puffed Grinning Nose Wrinkled Whistling	FNGS2.0	IDFNP (Inception v3 CNN + DeepID CNN)	2D images 860 FP patients	Accuracy 97.5%	
Muhammad Sajid et al. 2018 [5]	FP classification (5 grades)		HB	CNNs GAN	2D images 2000 Patients	Accuracy 92.60%	
Banita and Tanwar. 2018 [12]	Evaluation of FP 3 categories for patient (can be cured, cannot be cured, may or may not be cured)		HB	Fuzzy logic	3D images 82 patients		
Ting Wang et al. 2015 [11]	FP classification (6 grades)	Raise eyebrows Close eyes Screw up nose Plump cheeks Open mouth	HB	FPASMs SVM (RBF Kernel)	62 FP patients single-side and both-side		
Anguraj and Padma 2015 [13]	Classifying the severity of facial paralysis (normal–mild–moderate–severe)	Closing of eye Raising of eyebrows Opening of mouth Screwing of nose		SPSA FFBPN	9 images (2D and grayscale)	Accuracy 94% Sensitivity 90%	2D grayscale images Small number of images

CNNs: Convolutional Neural Networks, HB: House–Brackmann, LSCI: laser speckle contrast imaging, K-NN: K-nearest neighbor, SVM: Support Vector Machine, NN: Neural Network, PHCNN: Parallel Hierarchy Convolutional Neural Network, LSTM: Long Short-Term Memory, FNGS2.0: Facial Nerve Grading System 2.0, IDFNP: Inception-Deep Facial Nerve Paralysis, GAN: Generative Adversarial Network, FPASMs: Facial Paralysis Active Shape Models, RF: Random Forest, RLR: Regularized Logistic Regression, DT: Decision Tree, NB: Naïve Bayes, SPSA: Salient Point Selection Algorithm, FFBPN: Feed Forward Back Propagation Neural Network

### Limitations of computerized facial paralysis grading systems

Most of the present techniques are unable to cope with the most prevalent demanding conditions [14]. Wearing accessories is one of these challenges (e.g., glasses) in addition to the face’s odd appearance (mustache, haircut, etc.). Furthermore, the existing systems have numerous inter-personal variations in their outcomes. This means that the person cannot maintain the same expression all of the time.

The majority of studies in this area are based on datasets of 2D images [6, 13] with small number of cases and lack in severity levels variety. As a result, they have limited classification accuracy and hence, are not suitable for large-scale applications.

Face capture utilizing 2D imaging techniques has a number of drawbacks, including motions, occlusion between extremities, and lighting variations. In addition, external face asymmetry produced by position, orientation, illumination, and shadows [12].

Optical motion devices with reflective markers on the subject's face are utilized to capture 3D facial motions [15]. These systems are costly, and they require a professional clinician to place the markers in the proper locations. These markers may also cause patients to become uncomfortable and distort their facial movements.

In some studies, hand-crafted features methods are used to pick the suitable facial aspects for classifying the levels of FP [16, 17]. This may not be the best facial representation, leading to low performance evaluation.

Classifying approaches based on AdaBoost algorithm suffer from large sensitivity to noisy data [18]. Some recent studies use CNNs in classifying the degree of FP [1, 19]. Overfitting in results is an inherent limitation of CNNs. Augmentation of the dataset is essential to overcome overfitting caused while using CNNs [5].

There are some issues with systems that use the Kinect 1.0 to capture facial animation units (FAUs) [20]. This is due to the fact that only six FAUs are available, which is insufficient for upper and lower face characteristics [20]. In addition, the values of these six FAUs are unstable in real-time. Lip corners could also not be accurately tracked with Kinect 1.0. Furthermore, Kinect 1.0 was unable to capture minor differences in eye characteristics.

### Aim of the current study

The current study is part of a comprehensive FP evaluation system based on Artificial Intelligence (AI) and Machine learning (ML) approaches. The system uses the Kinect V2 and the SDK 2.0 (Microsoft, USA) to automatically extract facial landmarks and Facial Animation Units (FAUs) from FP patients. The evaluation system consists of two phases: FP assessment and FP classification.

The work presented in this paper focuses on the FP classification phase and is an extension of previous research by the authors [21]. The previous study presented a system to classify six normal facial functions: smiling, eye closure, raising the eyebrows, blowing cheeks, whistling, and resting.

The FP classification phase described in this paper classifies between right and left FP in three severity levels of paralysis: mild, moderate, and severe. This is performed for the five voluntary facial movements in addition to the resting state. An ensemble-based classifier with two learning levels is used. To the best of our knowledge, this methodology of FP classification has not been reported in the literature.

## Results

This section describes the results of testing and evaluating the FP classification module. This module is responsible for classifying the severity level of FP based on the resultant features acquired from the FP assessment stage. Seven severity categories were considered: left mild FP, left moderate FP, left severe FP, right mild FP, right moderate FP, right severe FP, and normal.

As an initial stage, five SVMs classifiers are considered, each classifier is trained on the features of a specific facial movement. In the second stage, the ensemble learning strategy is added to improve the prediction results based on the learning of more than one classifier.

### Single classifier

Five classifiers are developed, each single classifier learns on the features of one facial movement. SVMs, K-NN, and random forests classifiers were trained and tested to choose the best one with the highest performance.

**Fig. 1:**
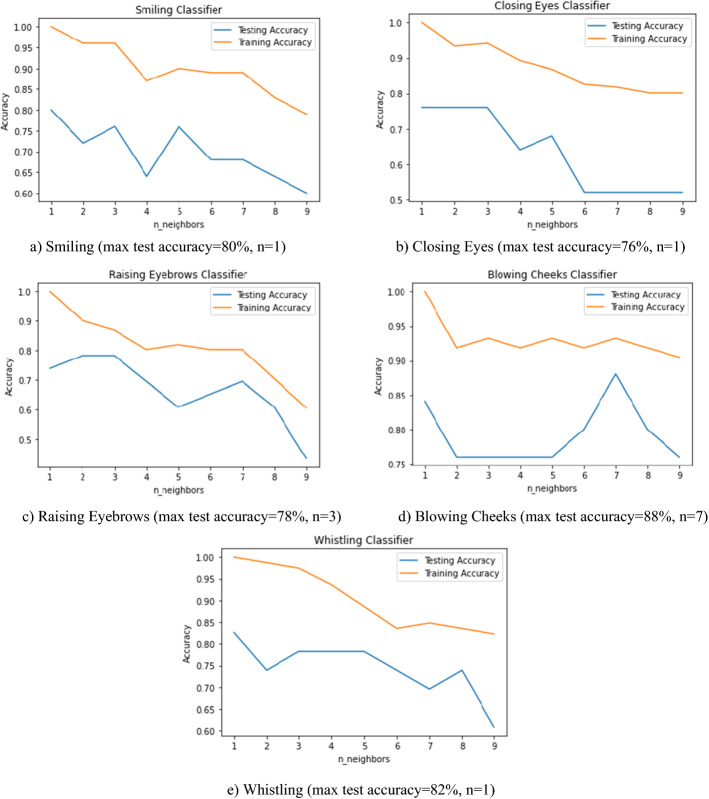
Variation of K-NN accuracies with changing the number of nearest neighbors parameter (from 1 to 9) in the five classifiers: **a** smiling, **b** closing eyes, **c** raising eyebrows, **d** blowing cheeks, and **e** whistling

Table 2 shows the maximum CV accuracy with its corresponding best values of C and gamma for the five SVM classifiers after undersampling but without data augmentation. Table 3 shows the maximum CV accuracy with its corresponding best values of hyperparameters (maximum depth and number of estimators) for the five random forests classifiers after undersampling but without data augmentation. Figure 1 shows the accuracies of each one of the five classifiers versus the number of nearest neighbors. The results show that SVMs classifiers have the maximum performances rather than Random Forests and K-NN.

**Table 2:** Maximum cross-validation accuracy and its corresponding best values of C and gamma for the five SVM classifiers (without data augmentation)

Classifier	#1 Smiling	#2 Closing eyes	#3 Raising eyebrows	#4 Blowing cheeks	#5 Whistling
Accuracy %	96	91	84	90	91
C	10^7^	100	10^7^	10^8^	100
Gamma	1	10	10	0.1	10

The ranges of C and gamma are (10^–3^, 10^–2^ ……. 10^8^) and (10^–3^, 10^–2^ ……. 10^3^), respectively

**Table 3:** Maximum cross-validation accuracy and its corresponding best values of hyperparameters for the five Random Forests classifiers (without data augmentation)

Classifier	#1 Smiling	#2 Closing eyes	#3 Raising eyebrows	#4 Blowing cheeks	#5 Whistling
Accuracy %	76	56	61	88	91
max_depth	6	8	8	8	10
n_estimators	100	30	100	50	30

The values of max_depth and n_estimators are (1, 2 …. 10) and (5, 10, 20, 30, 50,100), respectively

The performance of each individual SVM classifier was evaluated using the test set as shown in Table 4 in two cases: with and without threshold changes. Performance metrics are the accuracy, precision, sensitivity, F1-score, and specificity. Performance is shown for five SVM classifiers: smiling, closing eyes, raising eyebrows, blowing cheeks, and whistling). The performance of the single classifier (as shown in Table 2) was found to be not good enough to take the decision on the level of FP. This is because the severity level of FP is affected by the performance of all the five movements simultaneously and not just a single movement.

**Table 4:** Performance measure of the five individual SVM classifiers (with and without threshold change) and the ensemble-based classifier

Classifier	#1 Smiling		#2 Closing eyes		#3 Raising eyebrows		#4 Blowing cheeks		#5 Whistling		Ensemble
Threshold change?	No	Yes	No	Yes	No	Yes	No	Yes	No	Yes	Yes
Accuracy %	93.6	95.2	90.4	92	87.2	92	90.4	93.6	77.6	87.2	96.8
Precision %	88	92	80	84	72	84	80	88	48	72	96
Sensitivity %	81.5	85.2	74.1	77.8	66.7	77.8	74.1	81.5	44.4	66.7	88.9
F1-score %	84.6	88.5	76.9	80.8	69.2	80.8	76.9	84.6	46.2	69.2	92.3
Specificity %	96.9	98	94.9	95.9	92.9	95.9	94.9	96.9	86.7	92.9	99

### Ensemble-based classifier

To improve the performance results of classifying FP, a new ensemble-based classifier was developed. It combines the advantages of the bagging and stacking approaches. In addition, it is suitable for use with the high-dimensional feature space in the dataset.

The ensemble-based learning classifier involves two levels of classification: first level and second level. In the first level, the individual learners are the five SVMs classifiers used in parallel. The prediction results from the individual classifiers are then used as features to train the rule-based classifier in the second level. The resultant category predicted by the final classifier indicates the severity level of FP.

The performance of each individual classifier versus the performance of the new developed ensemble learning approach is shown in Table 4. The performance measures for each individual category using the ensemble learning classifier are shown in Table 5. The confusion matrix of the ensemble classifier is described in Table 6.

**Table 5:** Performance measure for each individual category using the ensemble-based classifier

Class	N	L_MI	R_MI	R_MO	R_S
Accuracy %	88	92	100	96	92
Sensitivity %	100	71.3	100	87.5	100
Specificity %	84.2	100	100	100	100

**Table 6:** Confusion Matrix for the ensemble classifier

Predicted class	N	L_MI	R_MI	R_MO	R_S
Normal (N)	**40**	0	0	0	0
Left Mild (L_MI)	10	**25**	0	0	0
Right Mild (R_MI)	0	0	**10**	0	0
Right Moderate (R_MO)	5	0	0	**35**	0
Right Severe (R_S)	0	0	0	0	**10**

The bold indicates the true predicted values

### Robustness and stability of the FP classifiers

In the case of using single classifiers (SVMs with RBF kernels), the model robustness is evaluated using fivefold CV in two cases: with and without applying data augmentation. The performances of the models (accuracy, F1-score, precision, and sensitivity) were computed five consecutive times with different splits each time. The average results and standard deviation (STD) of accuracy, precision, sensitivity, and F1-score were then calculated as shown in Table 7.

**Table 7:** SVM models performances measures using fivefold CV (with and without data augmentation)

Classifier	#1 Smiling		#2 Closing eyes		#3 Raising eyebrows		#4 Blowing cheeks		#5 Whistling	
Augmentation?	No	Yes	No	Yes	No	Yes	No	Yes	No	Yes
Accuracy %	90 ± 7	98 ± 1	86 ± 4	98 ± 1	73 ± 8	94 ± 3	82 ± 8	93 ± 3	87 ± 5	96 ± 4
Precision %	90 ± 10	98 ± 2	81 ± 9	98 ± 1	71 ± 15	94 ± 4	87 ± 5	93 ± 3	90 ± 6	95 ± 4
Sensitivity %	89 ± 11	98 ± 2	78 ± 10	98 ± 1	70 ± 10	93 ± 4	84 ± 5	93 ± 4	87 ± 4	96 ± 3
F1-score %	88 ± 12	98 ± 2	77 ± 9	98 ± 1	68 ± 14	93 ± 4	84 ± 5	93 ± 4	85 ± 6	95 ± 4

## Discussion

There is ongoing research in the field of classification and grading of FP, and this is because a fast, quantitative, objective and clinically feasible system is still needed. Current research by the authors involves designing, developing and testing a comprehensive automated assessment and classification system for FP. The work presented in this paper is the final module of this system, and is the module responsible for classifying the severity level of FP. Seven severity categories were considered: left mild, moderate and severe FP, right mild, moderate and severe FP as well as the normal.

The first stage of the work was selecting the facial features to be used and the method of extracting them. The majority of previous studies related to FP classification used two-dimensional dataset images which are affected by orientation and lighting [12]. In some studies, manual and thus subjective landmark detection was performed [15, 37]. Other studies use deep learning and specialized feature recognition software to extract features from 2D images [5]. For 3D facial capture, optical systems have been previously used [15]. However, these systems are expensive and need a specialized clinician to place markers on the face. These markers may disturb the patients and distort their facial movements.

The Kinect V2, with SDK 2.0, overcomes several of these limitations for 3D facial data acquisition. It is automatic, fast, accurate and eliminates the need for a specialized clinician or additional feature recognition software. The system uses depth images to extract FAUs and 3D landmarks, and the data show high performance even with unusual appearance of the face such as mustache or wearing accessories (e.g., glasses) unlike with other systems [14]. Also there is no need for markers on the face and hence no physical contact with the patient which is an advantage during the Covid-19 pandemic. Furthermore, the FAUs reflect the action units (AUs) which in turn separate facial expressions into separate components of facial muscle movement. Thus the FAUs were selected as a viable option for features in FP assessment and classification in this work. However, this posed the first challenge faced which was the unavailability of FP datasets with the FAUs as features. Second, nothing of the available research is based on similar methods to compare the results with. This challenge was addressed by creating a dataset of FP with FAUs as features. The dataset includes 375 records of 13 unilateral FP patients performing the six facial movements.

Another challenge is the imbalanced dataset due to the small number of the FP cases with respect to the large number of healthy cases. To overcome this problem and enhance the classification performance, a hybrid strategy of three different techniques was proposed. The strategy includes undersampling (see “Undersampling” section) and augmentation (see “Data augmentation” section) techniques in the preprocessing phase and threshold change in the post-processing phase (in “Post-processing” section).

Table 4 shows that the performance of the new developed ensemble classifier is better than the performance of each individual classifier. Also, it is shown that the performances of the classifiers were improved after using the threshold change technique after the learning process.

Table 7 shows that the performance of the model is better when using augmentation than without using augmentation. Also, the values of the standard deviations when using augmentation before training are less than the standard deviations without using augmentation. This means that augmentation of the data leads the model to be more robust and stable and perform well for different train and test samples.

The work presented in this paper to the best of our knowledge is unique in providing 5-categorical severity classification of paralysis. The procedure is quantitative, objective, and does not involve any discomfort.

This study includes some valuable contributions. One of the contributions is providing a comprehensive approach for FP evaluation including static and dynamic facial features. In addition, showing that the FAUs which are automatically extracted by the Kinect V2 may be employed as features in classifying FP. Furthermore, demonstrating a new ensemble learning technique for classifying seven severity levels of FP. Combining multiple classifiers in the ensemble learning gives higher prediction results (as shown in Table 4) than using just a single weak classifier. The newly developed ensemble approach was established for the high-dimensional data to gather the advantages of stacking and bagging.

The study’s limitations include the small number of FP patients tested and the lack of particular severity categories, such as left moderate FP and left severe FP. This is because the most prevalent cause of FP is the upper respiratory infection (e.g., influenza, cold). As a result, the number of FP patients rises at the start of the winter season and then drops during the remainder of the year. Another reason that has limited the number of FP patients visiting hospitals and clinics in the last two years is the corona virus pandemic.

The SVMs algorithm is the best choice for training as it is difficult to obtain a large enough training dataset of FP patients having different levels of severity. Also, applying data augmentation overcomes the lack of samples and enhances the performance of the classifier.

## Conclusion

This research is part of a comprehensive and automated framework for FP evaluation that is not invasive and provides accurate quantitative results. The current work describes the FP classification stage. A novel approach was proposed using the FAUs acquired by the Kinect sensor to automatically classify FP. The severity of FP is classified as one of seven categories: left mild FP, left moderate FP, left severe FP, right mild FP, right moderate FP, right FP, and normal. A new ensemble learning approach was developed based on SVMs as estimators to improve the prediction results rather than using just one weak classifier. The final decision is based on the developed rule-based classifier combining the results from the individual SVM classifiers. For handling the problem of imbalanced dataset, a hybrid strategy which consists of three different techniques was applied. Undersampling and data augmentation techniques were applied in the preprocessing phase, whereas changing the discriminating threshold was applied in the post-processing phase.

More FP patients with various FP severity levels should be considered. The work can be extended to assist in diagnosing patients having problems such as Alzheimer’s disease (AD) and autism.

## Methods

**Fig. 2:**
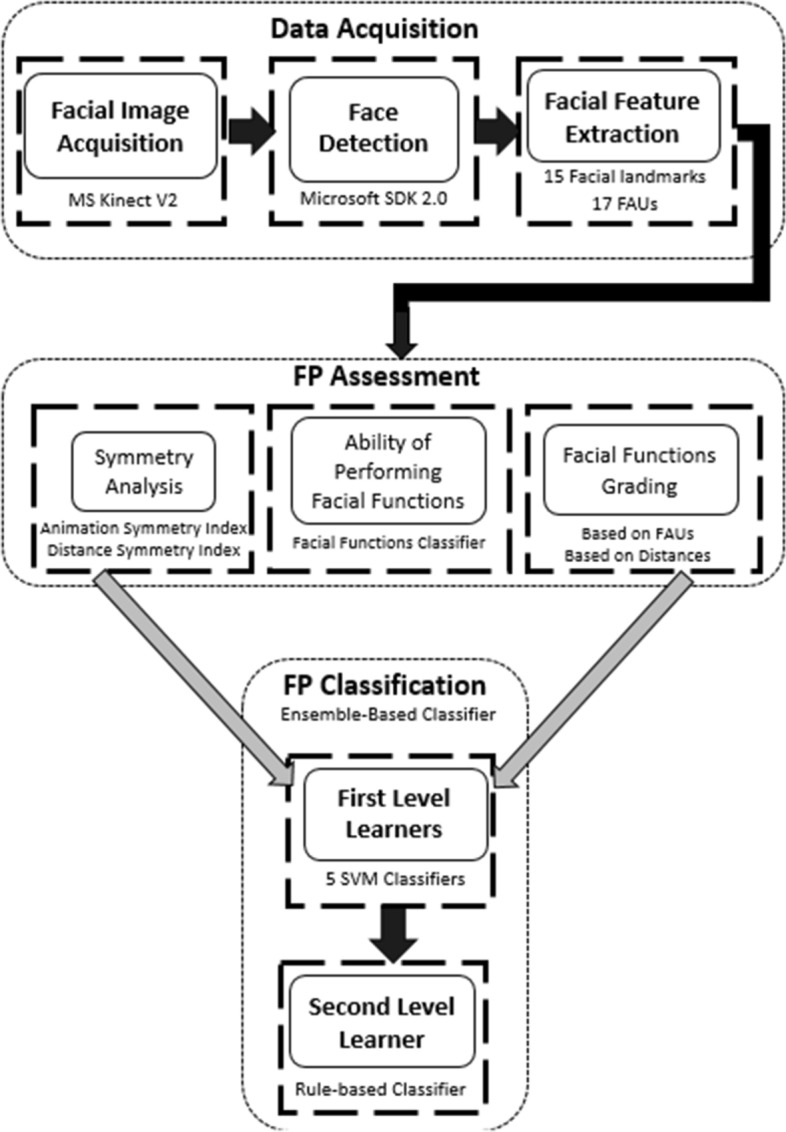
Block diagram of Facial Paralysis Evaluation system

The block diagram of the whole comprehensive FP evaluation system being developed is shown in Fig. 2. The FP classification module presented in this paper is used to classify three severity levels of both right and left unilateral FP. This is performed for five voluntary facial movements in addition to the resting state. The following sections describe building the dataset, feature selection and extraction followed by the feature processing and classification stages. The symmetry indices and degrees of performing the facial movements are computed from the FAUs and used as inputs to the classification module.

### Data acquisition

Patients were recruited for this study at the Al Kasr El Aini and Al Azhar hospitals, Cairo, Egypt. A total of 13 patients with various degrees of unilateral FP, mostly idiopathic, were included in the study. Characteristics of the patients are shown in Table 8. The characteristics include age, gender, paralyzed side, severity and duration of the condition. Each person sat on a 50-cm-high seat in a room with good lighting conditions, one meter away from the Kinect V2 sensor. The patients were instructed to perform five voluntary facial movements: raising eyebrows, closing eyes, smiling, blowing cheeks, and whistling. Data were captured during each of these movements in addition to the resting state. Several samples of each movement were acquired to build the FP dataset which includes a total of 375 records. These multiple samples of the same movement can be considered as samples from more patients. Sufficient resting time was allowed between successive samples from the same patient.

**Table 8:** Characteristics of FP patients

Patient #	Gender	Age (years)	Paralysis side	Duration of having FP (weeks)	Degree of paralysis	Type of paralysis
1	Female	30	Left	8	Mild	Chronic
2	Female	32	Left	9	Mild	Chronic
3	Female	40	Right	10	Moderate	Chronic
4	Female	38	Right	11	Moderate	Chronic
5	Male	17	Right	3	Moderate	Subacute
6	Male	16	Right	4	Moderate	Subacute
7	Male	18	Right	3	Mild	Subacute
8	Male	13	Left	12	Mild	Chronic
9	Female	55	Right	10	Moderate	Chronic
10	Female	60	Right	2	Severe	Acute
11	Male	52	Right	1	Severe	Acute
12	Female	58	Left	2	Mild	Acute
13	Female	50	Right	2	Severe	Acute

Fifty healthy participants were randomly recruited for this study. The participants’ ages ranged between 14 and 65 years. Subjects with any clear type of facial abnormality or asymmetry were excluded. Each subject was requested to perform the 5 movements. The normal dataset (previously developed by the authors [21]) includes a total of 1650 records of different states: resting, smiling, eye closure, eyebrows raising, cheeks blowing, and whistling.

The experimental procedures involving human subjects were approved by the ethics committee of the Systems and Biomedical Engineering department council, Cairo University. Participants involved in this research and parents of young patients were informed of the research procedures and signed an informed consent form.

The records acquired from the FP patients and normal subjects were combined in a complete dataset to be processed for the classification stage. Three levels of unilateral FP were considered in this study: mild, moderate and severe. The dataset was labeled accordingly by experienced clinicians and these labels are used as the ground truth. The dataset was then divided into seven categories: left mild FP, left moderate FP, left severe FP, right mild FP, right moderate FP, right severe FP, as well as the normal case. The symbols used for these categories and their frequencies in the dataset are shown in Table 9. As a proof of concept, only five classes were considered in this work: normal, left mild FP, right mild FP, right moderate FP and right severe FP due to the unavailability of the two classes: left moderate FP and left severe FP.

**Table 9:** Seven categories of FP classification and their frequencies in the dataset

Category	Description	Frequency
N	Normal	289
L_MI	Left mild facial paralysis	127
L_MO	Left moderate facial paralysis	0
L_S	Left severe facial paralysis	0
R_MI	Right mild facial paralysis	35
R_MO	Right moderate facial paralysis	177
R_S	Right severe facial paralysis	36

### Feature extraction

The Facial Action Coding System (FACS) is a system for characterizing facial muscles movements and how the appearance of the face changes with these movements [22]. Certain variations in the facial appearance are the result of several muscle movements, and some muscles can be involved in multiple actions. FACS analyzes each facial emotion into action units (AUs), which are separate components of facial muscles movements [23].

The SDK 2.0 for the Kinect V2 includes a library for automatically acquiring 3D facial landmarks and Facial Animation Units (FAUs) which reflect the AUs. 3D facial landmarks from the Kinect sensor have been used previously in facial functions’ assessment [24–28], and in FP evaluation [29]. FAUs from the Kinect sensor were previously used as a features for facial emotion and expression recognition [30–32].

**Fig. 3:**
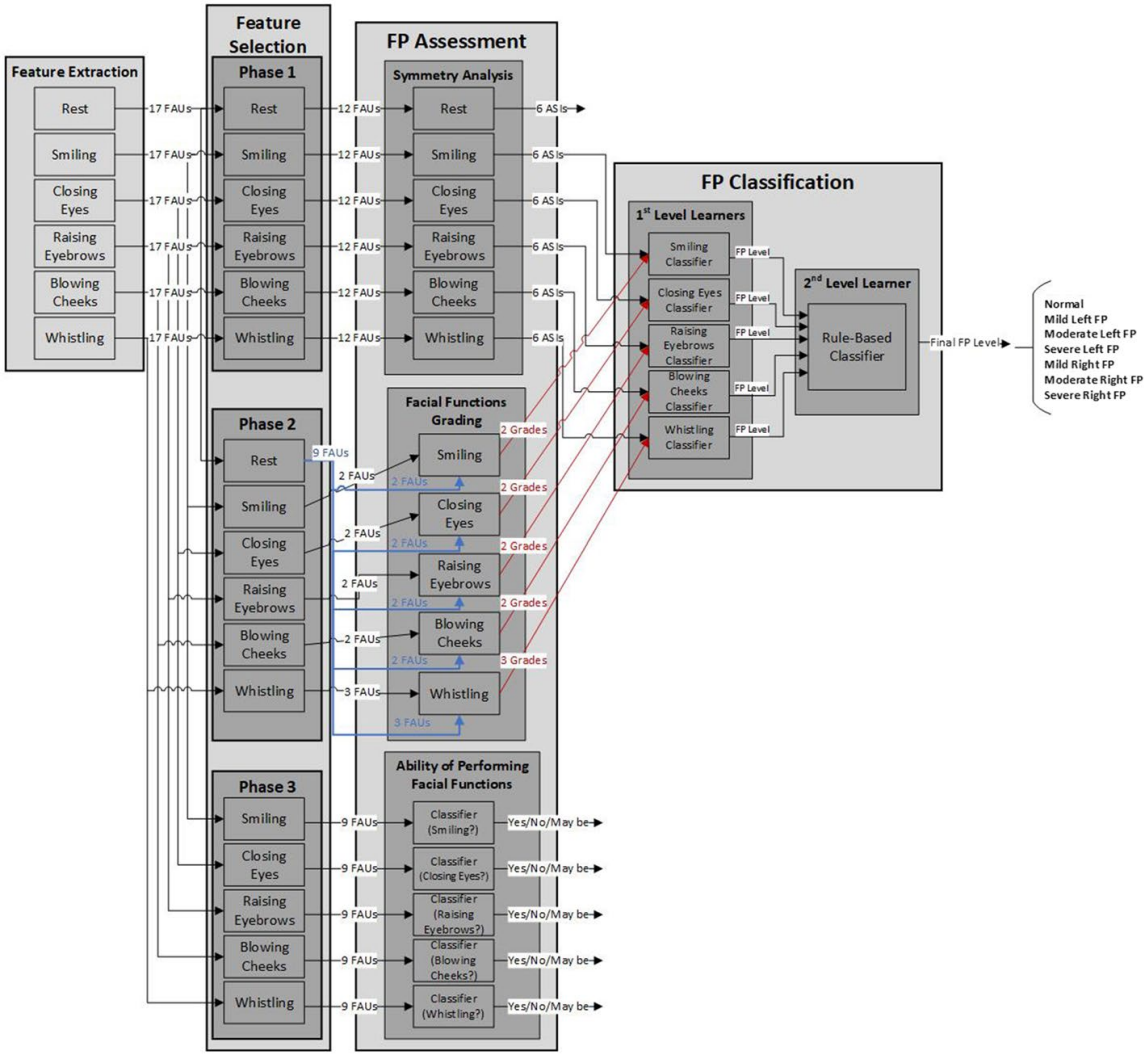
Detailed analysis of features in each stage of FP evaluation

In this study, seventeen FAUs are extracted from each FP patient during performing the six movements which include: rest, smiling, eye closure, eyebrows lifting, blowing cheeks, and whistling. Therefore 102 feature values per record are available to be used for classifying the degree and type of FP. The detailed analysis of features in the whole block diagram of the study is shown in Fig. 3. The figure shows the number and type of features used in each stage of FP evaluation starting from the data acquisition until reaching the final classification of FP severity level.

### Feature selection

As inputs to the two modules, symmetry analysis module, and facial functions grading module for feature transformation, two separate sets of FAUs were selected from the 102 FAUs available per record. The first set of features are the 12 FAUs: FAU2, FAU3, FAU4, FAU5, FAU6, FAU7, FAU8, FAU9, FAU10, FAU11, FAU16, and FAU17 for each one of the six facial movements. These features (72 FAUs) are then fed to the symmetry analysis module previously developed by the authors to calculate the animation symmetry indices (ASIs).

The second set of features includes the most affected FAUs involved in each one of the five facial movements as follows: (FAU2 and FAU3) in eye closure, (FAU4 and FAU5) in eyebrows lifting, (FAU6 and FAU7) in smiling, (FAU16 and FAU17) in cheeks blowing, and (FAU14, FAU16, and FAU17) in whistling. These eleven FAUs in addition to their corresponding ones (9 FAUs) in the resting state excluding the repetition of FAU16 and FAU17 (for whistling and blowing cheeks) form the second set of features. These 20 features are the inputs to the facial functions grading module previously developed by the authors to compute the degree of performing the facial movements.

Most of the features in the second set are included in the first set. Only 2 features (FAU14 in rest and whistling states) are in the second set and are not in the first set. Therefore, the total number of features used for these two modules are 74 FAUs.

### Pre-processing

#### Feature transformation

**Fig. 4:**
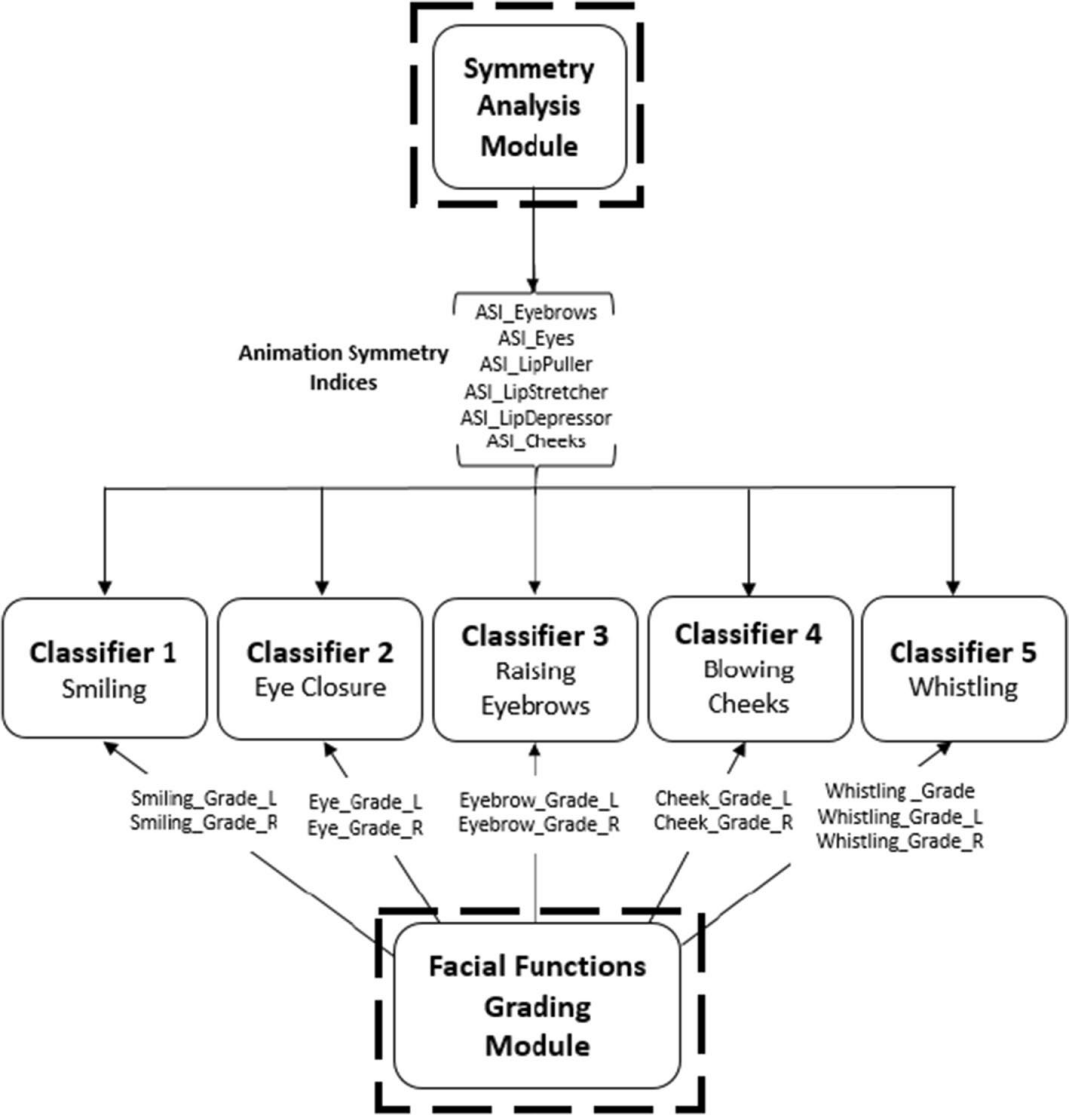
Framework of the classifiers and the corresponding features from the grading and symmetry modules

As described in Figs. 3 and 4, the FAUs selected are not used directly as inputs to the classification module. Two modules shown in Fig. 2 are used to transform the FAUs features into new sets of features.

In the symmetry analysis module, the values of FAUs are compared between the right and left sides of the face during the 6 movements to evaluate the animation symmetry indices (ASIs) of the three facial regions: mouth, eyes, and eyebrows. For each one of the six facial movements, six ASIs are computed from the 12 FAUs provided (as shown in Fig. 3). The total number of ASIs output from the symmetry analysis module is 36 ASIs. These features vary according to different facial movements, and thus the six ASIs evaluated while the subject is smiling, for instance, are fed to the smiling classifier (the classifier that classifies the severity and type of FP based on the smiling behavior).

In the facial functions grading module, the values of FAUs captured during each facial movement are compared to their corresponding ones in the rest state to evaluate the degree of achieving the movement. These features therefore indicate the grades of performing the facial movement for both sides of the face.

#### Undersampling

This problem of classification is an imbalanced domain learning problem in which the normal class is the majority class and contains a larger number of cases than the minority classes (FP classes). The training models are more likely to learn the majority class rather than the minority class (rare cases) [33]. Therefore, it is desired to bias the model to those rare classes (the classes of interest). A hybrid strategy which consists of three imbalanced learning techniques in two different phases, preprocessing phase and post-processing phase, was applied. Undersampling and data augmentation (in “Data augmentation” section) techniques were applied in the preprocessing phase while changing the discriminating threshold was applied in the post-processing phase (in “Post-processing” section).

Random undersampling is down-sampling the most represented and less important class by randomly removing samples. Although applying this technique is simple, useful data may be discarded. Also, this technique must be applied with caution in small data sets. In this study, random undersampling technique was applied on the normal class. The original number of samples acquired from normal subjects was 1650 [21]. After performing random undersampling, the number of normal samples was decreased to 289 samples as shown in Table 9. The total number of samples in the dataset becomes 664 samples (289 normal + 375 different FP cases).

#### Data augmentation

In this study, the minority classes (FP classes) are equally important in the prediction problem. Therefore, a data augmentation technique was applied for synthesizing new samples of the minority classes so that the number of samples in the minority classes better resembles or matches the number of samples in the majority class (normal class). Synthetic Minority Oversampling Technique (SMOTE) [34] is an oversampling technique, in which a new minority class sample is created between a randomly selected seed sample from that class and one of its K-nearest neighbors.

SMOTE is used to oversample all classes to have the same number of samples (i.e., 289 samples) as the class with the most samples. SMOTE is better than random oversampling as it works by creating synthetic samples from the minor classes instead of creating copies. One of the limitations of SMOTE appears when the minority class samples are very sparse which leads to a greater chance of class mixture.

### Data splitting and cross-validation

The acquired dataset was divided into training and validation sets and testing set with the ratio around 4:1. The training and validation set contain about 80% of the dataset and the other 20% was used for testing. The splitting of data was performed randomly, but stratified to make sure that all the classes appear in the training and testing data with the same distribution present in the dataset.

Fivefold cross-validation (CV) was used to divide the training and validation set into fivefolds. In such fivefold CV, one of the folds is left out as the validation data, whereas the remaining folds are used as the training data for model building. CV is a resampling procedure used to evaluate ML models on a limited data sample [37].

### Ensemble-based learning

Ensemble learning methods are algorithms that combine the results from more than one model. They are developed to improve the prediction results based on the learning of more than one classifier [35]. Different classifier combination approaches were developed such as bagging, stacking and boosting. Each has its own advantages and disadvantages.

**Fig. 5:**
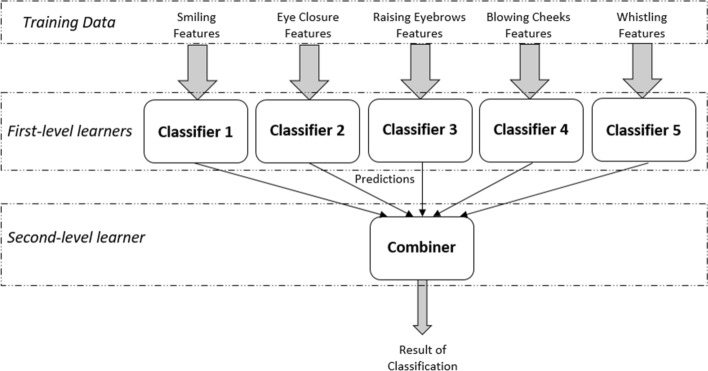
Framework of Facial Paralysis Classification approach

In this study, a new ensemble approach was developed to combine the advantages of the bagging and stacking algorithms to reduce the high-dimensional dataset. The framework of this approach is described in Fig. 5.

The classification process involves two phases of learning: first level and second level.

#### First-level learners

In the first level, five classifiers are employed and trained in parallel on the features from the five movements independently. Three different models were trained and tested in this stage: SVMs, K-NN, and random forests. The results showed that SVMs outperform K-NN and random forests. Therefore, SVMs are chosen as the individual classifiers in this level of learning. RBF Kernel models with their optimum parameters are used. Each classifier was trained with a subset of features acquired from a specific facial movement performed by the FP patient. The result from each classifier will be one of the five FP categories: normal, left mild FP, right mild FP, right moderate FP, and right severe FP.

The original feature space includes 36 ASIs output from the symmetry analysis module and eleven facial grades values output from the facial functions grading module. But only 30 ASIs (excluding the 6 ASIs from the rest state) are used for the classification with the 11 facial grades values. Therefore, a total of 41 features are used as inputs to the classification process. The dataset was partitioned to a subset of features. Each subset has the features corresponding to a certain facial movement. Six ASIs and two difference in FAUs from the rest values are considered for each one of the four facial movements: smiling, raising eyebrows, closing eyes, and blowing cheeks. Six ASIs and three difference in FAUs from the rest values are considered for whistling (as shown in Fig. 3).

#### Second-level learner

**Fig. 6:**
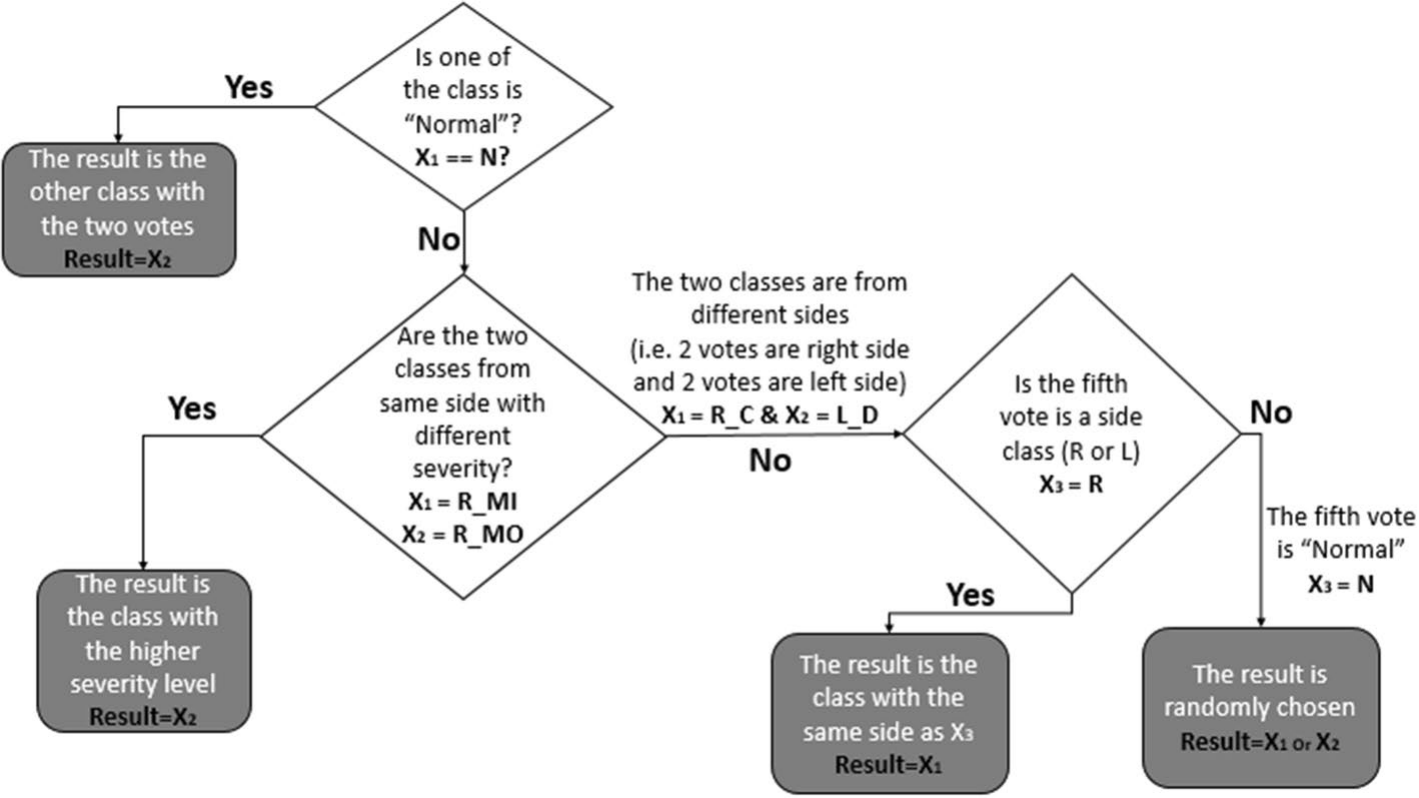
Flowchart of the rule-based classifier procedure

In the second level, the five prediction results from each classifier in the first phase are then combined and input to a rule-based classifier (the combiner) to make the final decision that is one of the five FP categories. The result of the classifier is initially based on the maximum vote criteria. However, when two classes have the same number of votes (i.e., two votes for each class), the result will then be based on other conditions as illustrated in Fig. 6.

The performance of each individual classifier and the performance of the new developed ensemble learning were evaluated and compared.

### Hyperparameters optimization

The performance of the SVMs kernels models is sensitive to the hyperparameter values. There are two parameters for an RBF kernel: C (cost) and gamma. Hyperparamters tuning was performed on a specified range of C and gamma values. Grid-search method was used with the appropriate ranges of values as follows: C (10^–3^, 10^–2^ ……. 10^8^), and gamma (10^–3^, 10^–2^ ……. 10^3^). Fivefold CV technique was used to estimate the accuracy of each parameter combination in the selected range to find the optimum values of C and gamma. This process is performed for each one of the 5 SVM classifiers in the first-level learning.

### Post-processing

As mentioned in “Undersampling” section, the other strategy used to overcome the problems in imbalanced learning is changing the discriminating threshold which is applied after the learning process [36]. Its goal is to manipulate the predictions of the models according to the domain preferences and the imbalance of the data. Adjusting the decision threshold is a good strategy to deal with the class imbalance problem.

After prediction, the probability estimate of the classes is used to set an appropriate value of the threshold to increase the model performance in classification. By default the SVM classifier predicts the sample with the class label which has the maximum probability value. Based on analyzing the initial results of the classifier, it was found that the probability of the normal class is above 0.4 if the actual class is normal. Otherwise, this probability is below 0.4 if the actual class is one of the FP classes. Therefore, a threshold value of 0.4 was set. If the normal class has the maximum probability but below 0.4, then it will be excluded from the classification and the sample will be labeled with the other class having the second rank of the probability value. Applying this strategy leads to increase in the classifier performance.

## Data Availability

All data generated or analyzed during this study are available from the corresponding author on reasonable request.
